# Summary of taxonomy changes ratified by the International Committee on Taxonomy of Viruses (ICTV) from the Animal dsRNA and ssRNA(−) Viruses Subcommittee, 2025

**DOI:** 10.1099/jgv.0.002112

**Published:** 2025-07-25

**Authors:** Holly R. Hughes, Matthew J. Ballinger, Yiming Bao, Nicolas Bejerman, Kim R. Blasdell, Thomas Briese, Julia Brignone, Jean Paul Carrera, Lander De Coninck, William Marciel de Souza, Humberto Debat, Ralf G. Dietzgen, Ralf Dürrwald, Mert Erdin, Anthony R. Fooks, Kristian M. Forbes, Juliana Freitas-Astúa, Jorge B. Garcia, Jemma L. Geoghegan, Rebecca M. Grimwood, Masayuki Horie, Timothy H. Hyndman, Reimar Johne, John D. Klena, Hideki Kondo, Eugene V. Koonin, Alexei Y. Kostygov, Mart Krupovic, Jens H. Kuhn, Michael Letko, Jun-Min Li, Yiyun Liu, Maria Laura Martin, Nathaniel Mull, Yael Nazar, Norbert Nowotny, Márcio Roberto Teixeira Nunes, Arnfinn Lodden Økland, Dennis Rubbenstroth, Brandy J. Russell, Eric Schott, Stephanie Seifert, Carina Sen, Elizabeth Shedroff, Tarja Sironen, Teemu Smura, Camila Prestes Dos Santos Tavares, Robert B. Tesh, Natasha L. Tilston, Noël Tordo, Nikos Vasilakis, Peter J. Walker, Fei Wang, Anna E. Whitfield, Shannon L.M. Whitmer, Yuri I. Wolf, Han Xia, Gong-Yin Ye, Zhuangxin Ye, Vyacheslav Yurchenko, Mingli Zhao, ICTV Taxonomy Summary Consortium, EM Adriaenssens

**Affiliations:** 1 Centers for Disease Control and Prevention, Fort Collins, USA; 2 Biological Sciences, Mississippi State University, Mississippi State, USA; 3 National Genomics Data Center, China National Center for Bioinformation; Beijing Institute of Genomics, Chinese Academy of Sciences; University of Chinese Academy of Sciences, Beijing, PR China; 4 Consejo Nacional de Investigaciones Científicas y Técnicas (CONICET) and Instituto Nacional de Tecnología Agropecuaria (INTA), Buenos Aires, Argentina; 5 CSIRO Health and Biosecurity, Geelong, Australia; 6 Center for Infection and Immunity, and Department of Epidemiology, Mailman School of Public Health, Columbia University, New York, USA; 7 Instituto Nacional de Enfermedades Virales Humanas Dr. Julio I. Maiztegui. INEVH -ANLIS, Monteagudo 2510, Pergamino 2700, Argentina; 8 Instituto Conmemorativo Gorgas de Estudios de la Salud, Panama City, Panama; 9 Division of Clinical and Epidemiological Virology, KU Leuven, Leuven, Belgium; 10 Department of Microbiology, Immunology and Molecular Genetics, University of Kentucky, Lexington, USA; 11 Instituto Nacional de Tecnología Agropecuaria (INTA), Buenos Aires, Argentina; 12 QAAFI, The University of Queensland, St Lucia, Australia; 13 Robert Koch Institut, Berlin, Germany; 14 Department of Virology, University of Helsinki, Helsinki, Finland; 15 Animal and Plant Health Agency (APHA), Surrey, UK; 16 Department of Biological Sciences, University of Arkansas, Fayetteville, USA; 17 Embrapa Cassava and Fruits, Cruz das Almas, BA, Brazil; 18 Department of Microbiology and Immunology, University of Otago, Dunedin, New Zealand; 19 Graduate School of Veterinary Science, Osaka Metropolitan University, Izumisano, Osaka, Japan; 20 Osaka International Research Center for Infectious Diseases, Osaka Metropolitan University, Osaka, Japan; 21 School of Veterinary Medicine, Murdoch University, Murdoch, WA, Australia; 22 German Federal Institute for Risk Assessment, Berlin, Germany; 23 Viral Special Pathogens Branch, The Centers for Disease Control and Prevention, 1600 Clifton Rd, Atlanta, GA 30329, USA; 24 Institute of Plant Science and Resources, Okayama University, Kurashiki 710-0046, Japan; 25 Computational Biology Branch, Division of Intramural Research National Library of Medicine, National Institutes of Health, Bethesda, Maryland, USA; 26 University of Ostrava, Ostrava, Czechia; 27 Institut Pasteur, Université Paris Cité, CNRS UMR6047, Archaeal Virology Unit, Paris, France; 28 Integrated Research Facility at Fort Detrick, National Institute of Allergy and Infectious Diseases, National Institutes of Health, Fort Detrick, Frederick, MD, USA; 29 Paul G. Allen School for Global Health, Washington State University, Pullman, USA; 30 Institute of Plant Virology, Ningbo University, Ningbo, PR China; 31 Department of Natural Sciences, Shawnee State University, Portsmouth, USA; 32 Department of Biological Sciences and Pathobiology, University of Veterinary Medicine Vienna, Vienna, Austria; 33 College of Medicine, Mohammed Bin Rashid University of Medicine and Health Sciences, Dubai Health, Dubai, UAE; 34 Universidade Federal do Pará, Bélem, Brazil; 35 Pharmaq Analytiq, Bergen, Norway; 36 Institute of Diagnostic Virology, Friedrich-Loeffler-Institut, Greifswald – Riems, Germany; 37 Institute of Marine and Environmental Technology, University of Maryland Center for Environmental Science, Baltimore, Maryland, USA; 38 Integrated Group of Aquaculture and Environmental Studies, Federal University of Paraná, Curitiba, Paraná, Brazil; 39 Department of Pathology, The University of Texas Medical Branch, Galveston, TX, USA; 40 Department of Microbiology and Immunology, Indiana University School of Medicine, Indianapolis, USA; 41 Institut Pasteur, Conakry, Guinée; 42 University of Queensland, St Lucia, Australia; 43 Wuhan Institute of Virology, Chinese Academy of Sciences, Wuhan, PR China; 44 North Carolina State University, Raleigh, NC, USA; 45 Institute of Insect Sciences, Zhejiang University, Hangzhou, PR China; 46 Department of Pathobiology and Population Sciences, Royal Veterinary College, London, UK

**Keywords:** *Alphahymrhavirus ectemnius*, *Alphahymrhavirus electrico*, *Alphanemrhavirus wufeng*, *Alphapaprhavirus gata*, *Alphapaprhavirus orgi*, *Alphaplatrhavirus*, *Alphaplatrhavirus acutispina*, *Alphaplatrhavirus chodsigoa*, *Alphaplatrhavirus dendriticum*, *Alphaplatrhavirus hubei*, *Alphaplatrhavirus jingmen*, *Alphaplatrhavirus langier*, *Alphaplatrhavirus larvatus*, *Alphaplatrhavirus microphallus*, *Alphaplatrhavirus nodulosis*, *Alphaplatrhavirus orientalis*, *Alphaplatrhavirus pseudoglobulus*, *Alphaplatrhavirus ricketti*, *Alphaplatrhavirus smithii*, *Alphaplatrhavirus solidus*, *Alphaplatrhavirus turkestanicum*, *Alphaplatrhavirus vulpes*, *Alphaplatrhavirus wenzhou*, *Alphaplatrhavirus wufeng*, *Alpharicinrhavirus heilongjiang*, *Alphathriprhavirus*, *Antennavirus trematomi*, *Artemvirus*, *Artemvirus bsaeighalis*, *Artemvirus bsafalis*, *Artemvirus bsafialis*, *Artemvirus bsafivalis*, *Artemvirus bsasecalis*, *Artemvirus bsasialis*, *Artemvirus bsathalis*, *Betaplatrhavirus*, *Betaplatrhavirus abramus*, *Betaplatrhavirus armiger*, *Betaplatrhavirus beihai*, *Betaplatrhavirus fujian*, *Betaplatrhavirus fuscus*, *Betaplatrhavirus himastelon*, *Betaplatrhavirus nodulosis*, *Betaplatrhavirus pseudoglobulus*, *Betaplatrhavirus psilotrema*, *Betaplatrhavirus simillimum*, *Betaplatrhavirus sphaeroidotrema*, *Betaplatrhavirus wenling*, *Betathriprhavirus*, *Betathriprhavirus midwest*, *Betathriprhavirus variabilis*, *Canmovirus*, *Canmovirus mahaense*, *Cardoreovirus callinectes*, *Coroavirus*, *Coroavirus germense*, *Crabreovirus*, *Crabreovirus callinectes*, *Crabreovirus eriocheiris*, *Crabreovirus scylla*, *Cultervirus harpadoni*, *Cultervirus poeciliae*, *Dianlovirus dehongense*, *Feravirus hemipterus*, *Gammaplatrhavirus*, *Gammaplatrhavirus beihai*, *Gammaplatrhavirus dendriticum*, *Gammaplatrhavirus jilin*, *Gammaplatrhavirus orientalis*, *Gammaplatrhavirus sinensis*, *Gammaplatrhavirus wenzhou*, *Hexartovirus artemiae*, *Hexartovirus caligi*, *Jonvirus mikadosis*, *Jonvirus spilikinsis*, *Mammarenavirus vello*, *Merhavirus cambodia*, *Merhavirus subalbatus*, *Negarnaviricota*, *Orthobornavirus iridiscincum*, *Orthobunyavirus indianense*, *Orthobunyavirus lichuanense*, *Orthobunyavirus taiense*, *Orthobunyavirus taniyamense*, *Orthohantavirus ozarkense*, *Orthohantavirus sagercreekense*, *Orthophasmavirus barstukasense*, *Orthophasmavirus barstukorius*, *Orthophasmavirus coleopterus*, *Orthophasmavirus flenense*, *Orthophasmavirus miglotalis*, *Orthophasmavirus miglotasense*, *Orthophasmavirus obscurae*, *Orthophasmavirus stecellulae*, *Peropuvirus pteropi*, *Peropuvirus wufengense*, *Platrhavirus*, *Platrhavirus microphallus*, *Platrhavirus nodulosis*, *Platrhavirus orientalis*, *Platrhavirus pseudoglobulus*, *Platrhavirus turkestanicum*, *Platrhavirus vulpes*, *Polyploviricotina*, *Primrhavirus yunnan*, *Robevirus*, *Robevirus hanzense*, *Rotavirus kappaenteritidis*, *Rotavirus lambdaenteritidis*, *Shilevirus alphablechomonadis*, *Shilevirus alphamoraviense*, *Shilevirus betablechomonadis*, *Shilevirus betamoraviense*, *Shilevirus crithidiaebombi*, *Shilevirus gammablechomonadis*, *Shilevirus martiniquense*, *Shilevirus moramangoense*, *Shilevirus otongatchiense*, *Shilevirus puertonapoense*, *Sigmavirus dorsalis*, *Sigmavirus hangzhou*, *Stangrhavirus yunnan*, *Thriprhavirus*, *Tupavirus wufeng*, *Weflthvirus*, *Weflthvirus itaense*

## Abstract

RNA viruses are ubiquitous in the environment and are important pathogens of humans, animals and plants. In 2024, the International Committee on Taxonomy of Viruses Animal dsRNA and ssRNA(−) Viruses Subcommittee submitted 18 taxonomic proposals for consideration. These proposals expanded the known virosphere by classifying 9 new genera and 88 species for newly detected virus genomes. Of note, newly established species expand the large family of *Rhabdoviridae* to 580 species. A new species in the family *Arenaviridae* includes a virus detected in Antarctic fish with a unique split nucleoprotein ORF. Additionally, four new species were established for historically isolated viruses with previously unsequenced genomes. Furthermore, three species were abolished due to incomplete genome sequence information, and one family was moved from being unassigned in the phylum *Negarnaviricota* into a subphylum and order. Herein, we summarize the 18 ratified taxonomic proposals and the general features of the current taxonomy, thereby supporting public and animal health responses.

## Introduction

RNA viruses are widely distributed and infect a broad variety of hosts. As technological advancements in high-throughput sequencing and data analysis have exponentially expanded in the twenty-first century, so has the RNA virome [1]. The International Committee on Taxonomy of Viruses (ICTV) Animal dsRNA and ssRNA(−) Viruses Subcommittee (SC) was established in 2014 to develop a taxonomy for RNA viruses detected in the kingdom Animalia. Study Groups within the Subcommittee are responsible for many viral families in the phyla *Negarnaviricota* (orders: *Muvirales*, *Jingchuvirales*, *Mononegavirales*, *Goujianvirales*, *Elliovirales*, *Hareavirales* and *Articulavirales*) and *Duplornaviricota* (orders: *Reovirales* and *Ghabrivirales*) and the realm *Ribozyviria*.

The phylum *Negarnaviricota,* the largest taxon falling within the SC’s remit, comprises viruses that predominantly have ssRNA(−) genomes, though some have an ambisense coding arrangement. Animal viruses assigned to this phylum exhibit a diversity of genome organizations (e.g. non-segmented or containing two to eight segments, linear or circular); however, all viruses within this phylum encode homologous RNA-directed RNA polymerases (RdRP) that form a strongly supported clade in the RdRP phylogenetic tree of the kingdom *Orthornavirae* [2]. The subphylum *Haploviricotina* is distinguished by viruses that encode an RdRP with mRNA capping activity [3]. Families such as *Artoviridae*, *Bornaviridae*, *Filoviridae*, *Lispiviridae* and *Rhabdoviridae* are classified within this subphylum in the order *Mononegavirales*. Families such as *Hantaviridae*, *Peribunyaviridae*, *Phasmaviridae*, *Arenaviridae* and *Leishbuviridae* are classified in the subphylum *Polyploviricotina*, distinguished by viruses that have an RdRP with cap-snatching activity [3] and include the orders *Elliovirales*, *Hareavirales* and *Articulavirales*.

*Tosoviridae* includes a single species for a virus isolated from sea turtles [4]. This virus has a bi-segmented ssRNA(−) genome similar to certain polyploviricotines. However, phylogenetic analysis cannot definitively place this family in a subphylum, and so it is classified as an unassigned negarnaviricot.

The phylum *Duplornaviricota* includes viruses that possess dsRNA genomes and can be further characterized by the presence of an unusual *T*=1 capsid [5]. Viruses in this phylum that infect animals have genomes that are either non-segmented or have 9 to 12 segments. Viruses of the family *Sedoreoviridae* (order: *Reovirales*) have genomes of 10–12 dsRNA segments and virions that have a characteristic ‘smooth’ appearance [6] in contrast to viruses in the reoviral family, *Spinareoviridae*.

This summary includes the ratified taxonomic proposals from 2024 for the Animal dsRNA and ssRNA(−) Viruses SC and is not a comprehensive summary of all taxonomy proposals for dsRNA and ssRNA(−) viruses since some are covered by the Plant Viruses SC [7] and Fungal and Protist Viruses SC [8].

A file including all the Tables of taxonomic changes below is available as a supplementary file to this article.

## Main Text

## Contents

2024.001M.Alpharhabdovirinae_1ng_11nsp
2024.002M.Antennavirus_1nsp
2024.003M.Artoviridae_4nsp
2024.004M.Bornaviridae_3nsp
2024.005M.Cardoreovirus_1nsp
2024.006M.Deltarhabdovirinae_4nsp
2024.007M.Filoviridae_1nsp
2024.008M.Lispiviridae_5ngen_11nsp
2024.009M.Mammarenavirus_1nsp
2024.010M.Orthobunyavirus_4nsp
2024.011M.Orthohantavirus_1nsp
2024.012M.Orthohantavirus_1nsp
2024.013M.Phasmaviridae_4nsp_3ab_2rn
2024.014M.Platrhavirus_2ng_30nsp
2024.015M.Rotavirus_2nsp
2024.016M.Sedoreoviridae_1ng_3nsp
2024.017M.Shilevirus_10nsp
2024.018M.Tosoviridae_move

## 2024.001M.Alpharhabdovirinae_1ng_11nsp

**Title:** In the subfamily *Alpharhabdovirinae*, create nine new species in six existing genera (*Alphapaprhavirus*, *Sigmavirus*, *Merhavirus*, *Tupavirus*, *Alphanemrhavirus*, *Alpharicinrhavirus*), rename the existing genus *Thriprhavirus* (as *Alphathriprhavirus*), and create the new genus *Betathriprhavirus* including two new species (*Mononegavirales*: *Rhabdoviridae*)

**Authors:** Walker PJ (peter.walker@uq.edu.au), Bejerman N, Blasdell KR, Debat H, Dietzgen RG, Fooks AR, Freitas-Astúa J, Ramos-González PL, Kondo H, Kurath G, Shi M, Tesh RB, Tordo N, Vasilakis N, Whitfield AE

**Summary**

**Taxonomic rank(s) affected**
Genus and species (*Mononegavirales*: *Rhabdoviridae*: *Alpharhabdovirinae*)
**Description of current taxonomy**
The subfamily *Alpharhabdovirinae* currently comprises 33 genera and 235 species.
**Proposed taxonomic change(s)**
Create nine new species in six existing genera (*Alphapaprhavirus*, *Sigmavirus*, *Merhavirus*, *Tupavirus*, *Alphanemrhavirus* and *Alpharicinrhavirus*) for viruses recently detected in bats, shrew or various invertebrates by metagenomic sequencing. Rename the existing genus *Thriprhavirus* (as *Alphathriprhavirus*), and create a new genus *Betathriprhavirus* including two new species for viruses detected in thrips by metagenomic sequencing.
**Justification**
The viruses cluster phylogenetically with others in the existing or proposed genera in maximum likelihood trees inferred using L protein sequences. All new species in existing genera meet established demarcation criteria. The proposed renamed and new genera for viruses detected in thrips are well-separated phylogenetically.
**Submitted:** 09/06/24

**Table 1.** *Alpharhabdovirinae*, 12 new taxa*

**Table:** 

Operation	Rank	New taxon name	Exemplar	Accession
New taxon	Genus	*Betathriprhavirus*		
New taxon	Species	*Betathriprhavirus variabilis*	soybean thrips rhabdo-like virus 1	MT224147
New taxon	Species	*Betathriprhavirus midwest*	soybean thrips rhabdo-like virus 2	MT224148
New taxon	Species	*Alphapaprhavirus gata*	Gata virus	KX852388
New taxon	Species	*Alphapaprhavirus orgi*	Orgi virus	KX852386
New taxon	Species	*Sigmavirus hangzhou*	Hangzhou rhabdovirus 4	MZ209737
New taxon	Species	*Sigmavirus dorsalis*	Bactrocera dorsalis sigmavirus	MN745080
New taxon	Species	*Tupavirus wufeng*	Wufeng bat tupavirus 2	OQ715690
New taxon	Species	*Alpharicinrhavirus heilongjiang*	Tahe rhabdovirus 2	ON408171
New taxon	Species	*Merhavirus subalbatus*	Armigeres subalbatus rhabdovirus	LC775065
New taxon	Species	*Merhavirus cambodia*	Cambodia Anophales rhabdovirus	OR479699
New taxon	Species	*Alphanemrhavirus wufeng*	Wufeng shrew rhabdovirus 1	OQ715689

**Table 2.** *Alpharhabdovirinae*, one rename taxon*

**Table:** 

Operation	Rank	New taxon name	Previous taxon name
Rename taxon	Genus	*Alphathriprhavirus*	*Thriprhavirus*

*Source/full text: https://ictv.global/ictv/proposals/2024.001M.Alpharhabdovirinae_1ng_11nsp.zip.

## 2024.002M.Antennavirus_1nsp

**Title:** Create one new species in genus *Antennavirus* (*Hareavirales*; *Arenaviridae*)

**Authors:** Grimwood RG (rebecca.grimwood@postgrad.otago.ac.nz), Geoghegan JL, Kuhn JH

**Summary**

**Taxonomic rank(s) affected**
*Hareavirales*: *Arenaviridae*: *Antennavirus*
**Description of current taxonomy**
There are currently three recognized species in the genus *Antennavirus*.
**Proposed taxonomic change(s)**
Establishment of one new species in genus *Antennavirus* for Ross Sea rockcod virus, identified in a scaly rockcod (*Trematomus loennbergii* Regan, 1913) and a slender scalyhead (*Trematomus lepidorhinus* (Paul Pappenheim, 1911)) from the Ross Sea, Antarctica.
**Submitted:** 06/06/24.

**Table 3.** *Antennavirus*, one new taxon*

**Table:** 

Operation	Rank	New taxon name	Exemplar	Accession
New taxon	Species	*Antennavirus trematomi*	Ross Sea rockcod virus	L: PP590693; M: PP590768; S: PP590769

*Source/full text: https://ictv.global/ictv/proposals/2024.002M.Antennavirus_1nsp.zip.

## 2024.003M.Artoviridae_4nsp

**Title:** Create two new species in genus *Peropuvirus* and two new species in genus *Hexartovirus* (*Mononegavirales*: *Artoviridae*)

**Authors:** Økland, AL (arnfinn.lodden.okland@zoetis.com), Kuhn, J, Ye, G, Vasilakis, N

**Summary**

**Taxonomic rank(s) affected**
Species
**Description of current taxonomy**
The family *Artoviridae* currently includes two genera, *Hexartovirus* (two species) and *Peropuvirus* (seven species).
**Proposed taxonomic change(s)**
Create two new species in genus *Hexartovirus* and two new species in genus *Peropuvirus*.
**Justification**
The viruses proposed to be assigned to novel species have a minimum amino acid divergence of 44 % in their L proteins compared to classified family members and occupy different ecological niches.
**Submitted:** 21/06/2024

**Table 4.** *Artoviridae*, four new taxa*

**Table:** 

Operation	Rank	New taxon name	Exemplar	Accession
New taxon	Species	*Peropuvirus pteropi*	bat faecal-associated arto-like virus 2	ON872573
New taxon	Species	*Peropuvirus wufengense*	Wùfēng shrew peropuvirus 1	OQ715590
New taxon	Species	*Hexartovirus caligi*	Caligus clemensi hexartovirus 1	MZ484467
New taxon	Species	*Hexartovirus artemiae*	brine shrimp artovirus 1	OL472418

*Source/full text: https://ictv.global/ictv/proposals/2024.003M.Artoviridae_4nsp.zip.

## 2024.004M.Bornaviridae_3nsp

**Title**: Create three new species in the family *Bornaviridae* (*Mononegavirales*)

**Authors:** Briese T, Dürrwald R, Horie M, Hyndman TH, Jiménez-Clavero MA, Kuhn JH, Nowotny N, Pfaff F (florian.pfaff@fli.de), Rubbenstroth D, Tomonaga K

**Summary**

**Taxonomic rank(s) affected**
Genus (*Cultervirus*, *Orthobornavirus*)
**Description of current taxonomy**
*Riboviria*: *Orthornavirae*: *Negarnaviricota*: *Haploviricotina*: *Monjiviricetes*: *Mononegavirales*: *Bornaviridae*: *Cultervirus* (three species) and *Orthobornavirus* (nine species).
**Proposed taxonomic change(s)**
Add two new species to genus *Cultervirus* (*Cultervirus harpadoni*, *Cultervirus poeciliae*) and add one new species to genus *Orthobornavirus* (*Orthobornavirus iridiscincum*).
**Justification**
The proposed new species are based on newly released genome sequences in GenBank that meet the current bornavirid species demarcation criteria.
**Submitted:** 21/06/24; **Revised:** 20/09/24

**Table 5.** *Bornaviridae*, three new taxa*

**Table:** 

Operation	Rank	New taxon name	Exemplar	Accession
New taxon	Species	*Cultervirus poeciliae*	Pará molly bornavirus	BK063657
New taxon	Species	*Cultervirus harpadoni*	Bombay duck fish bornavirus	BK063658
New taxon	Species	*Orthobornavirus iridiscincum*	Carlia munda bornavirus	PP711183

*Source/full text: https://ictv.global/ictv/proposals/2024.004M.Bornaviridae_3nsp.zip.

## 2024.005M.Cardoreovirus_1nsp

**Title:** Create one new species in the genus *Cardoreovirus* (*Reovirales*: *Sedoreoviridae*)

**Authors:** Zhao M (mzhao@rvc.ac.uk), Schott E (schott@umces.edu), Tavares C

**Summary**

**Taxonomic rank(s) affected**
*Cardoreovirus* genus
**Description of current taxonomy**
The genus *Cardoreovirus* currently has only one species, *Cardoreovirus eriocheiris*, whose exemplar member is Eriocheir sinensis reovirus (EsRV).
**Proposed taxonomic change(s)**
A new species (*Cardoreovirus callinectes*) belonging to the *Cardoreovirus* genus should be established.
**Justification**
The exemplar virus (Callinectes sapidus reovirus 2, CsRV2) of the proposed new species – *Cardoreovirus callinectes* – exhibits amino acid sequence similarities ranging from 46 to 79% for segments 1–12 compared to EsRV in the established species *Cardoreovirus eriocheiris*. The maximum likelihood phylogenetic tree indicates that CsRV2 falls on a different branch but within the same clade as EsRV, suggesting that a new species should be classified within the *Cardoreovirus* genus.
**Submitted:** 14/06/24

**Table 6.** *Cardoreovirus*, one new taxon*

**Table:** 

Operation	Rank	New taxon name	Exemplar	Accession
New taxon	Species	*Cardoreovirus callinectes*	Callinectes sapidus reovirus 2	MW208677; MW208678; MW208679; MW208680; MW208681; MW208682; MW208683; MW208684; MW208685: MW208686; MW208687; MW208688

*Source/full text: https://ictv.global/ictv/proposals/2024.005M.Cardoreovirus_1nsp.zip.

## 2024.006M.Deltarhabdovirinae_4nsp

**Title:** In the subfamily *Deltarhabdovirinae*, create one new species in the genus *Stangrhavirus*, one new species in the genus *Primrhavirus* and two new species in the genus *Alphahymrhavirus*

**Authors:** Walker PJ (peter.walker@uq.edu.au), Bejerman N, Blasdell KR, Debat H, Dietzgen RG, Fooks AR, Freitas-Astúa J, Ramos-González PL, Kondo H, Kurath G, Shi M, Tesh RB, Tordo N, Vasilakis N, Whitfield AE

**Summary**

**Taxonomic rank(s) affected**
Species (*Mononegavirales*: *Rhabdoviridae*: *Deltarhabdovirinae*)
**Description of current taxonomy**
The subfamily *Deltarhabdovirinae* currently comprises 11 genera including 34 species for viruses detected in various invertebrates (arthropods, nematodes and crustaceans).
**Proposed taxonomic change(s)**
Create four new species in the subfamily *Deltarhabdovirinae*, one in the genus *Stangrhavirus* for a virus detected in mosquitoes, one in the genus *Primrhavirus* for a virus detected in mosquitoes and two in the genus *Alphahymrhavirus* for viruses detected in ants and wasps.
**Justification**
The viruses cluster phylogenetically with others in the existing genera in ML trees inferred using L protein sequences. All new species meet established demarcation criteria for the genera.
**Submitted:** 09/06/24

**Table 7.** *Deltarhabdovirinae*, four new taxa*

**Table:** 

Operation	Rank	New taxon name	Exemplar	Accession
New taxon	Species	*Stangrhavirus yunnan*	Xiangyun mono-chu-like virus 11	OL700136
New taxon	Species	*Primrhavirus yunnan*	Xiangyun mono-chu-like virus 4	OL700129
New taxon	Species	*Alphahymrhavirus electrico*	electric ant rhabdovirus	OP518027
New taxon	Species	*Alphahymrhavirus ectemnius*	Ectemnius rhabdovirus	BK063699

*Source/full text: https://ictv.global/ictv/proposals/2024.006M.Deltarhabdovirinae_4nsp.zip.

## 2024.007M.Filoviridae_1nsp

**Title:** Create one new species in the genus *Dianlovirus* (*Mononegavirales*: *Filoviridae*)

**Authors:** Kuhn, JH (kuhnjens@mail.nih.gov), Liu, Y, Bao, Y

**Summary**

**Taxonomic rank(s) affected**
Genus (*Dianlovirus*)
**Description of current taxonomy**
*Riboviria*: *Orthornavirae*: *Negarnaviricota*: *Haploviricotina*: *Monjiviricetes*: *Mononegavirales*: *Filoviridae*: *Dianlovirus*: *Dianlovirus menglaense*
**Proposed taxonomic change(s)**
Add one species (*Dianlovirus dehongense*)
**Justification**
The complete genome sequence of Déhóng virus (DEHV) fulfils the pairwise-sequence-based demarcation criterion for the establishment of a novel species.
**Submitted:** 21/06/24; **Revised:** 17/09/24

**Table 8.** *Filoviridae*, one new taxon*

**Table:** 

Operation	Rank	New taxon name	Exemplar	Accession
New taxon	Species	*Dianlovirus dehongense*	Déhóng virus	OP924273

*Source/full text: https://ictv.global/ictv/proposals/2024.007M.Filoviridae_1nsp.zip.

## 2024.008M.Lispiviridae_5ngen_11nsp

**Title:** Create five new genera and eleven new species in the family *Lispiviridae* (*Mononegavirales*)

**Authors:** Li JM (lijunmin@nbu.edu.cn), Ye GY, Wang F, Ye ZX

**Summary**

**Taxonomic rank(s) affected**
Mononegaviral family *Lispiviridae*.
**Description of current taxonomy**
Currently, the family *Lispiviridae* includes 25 genera and 34 species
**Proposed taxonomic change(s)**
We propose the creation of 5 new genera and 11 new species to be included in the mononegaviral family *Lispiviridae*.
**Justification**
Genus (and species) demarcation is proposed to be based on coding-complete genome sequence analyses, phylogenetic analyses and pairwise sequence comparisons similar to established genus/species demarcation criteria for other mononegaviral families.
**Submitted:** 05/06/24; **Revised:** 04/07/24

**Table 9.** *Lispiviridae*, 16 new taxa*

**Table:** 

Operation	Rank	New taxon name	Exemplar	Accession
New taxon	Genus	*Artemvirus*		
New taxon	Species	*Artemvirus bsafialis*	brine shrimp arlivirus 1	OL472403
New taxon	Species	*Artemvirus bsasecalis*	brine shrimp arlivirus 2	OL472404
New taxon	Species	*Artemvirus bsathalis*	brine shrimp arlivirus 3	OL472405
New taxon	Species	*Artemvirus bsafalis*	brine shrimp arlivirus 4	OL472406
New taxon	Species	*Artemvirus bsafivalis*	brine shrimp arlivirus 5	OL472407
New taxon	Species	*Artemvirus bsasialis*	brine shrimp arlivirus 6	OL472411
New taxon	Species	*Artemvirus bsaeighalis*	brine shrimp arlivirus 8	OL472416
New taxon	Genus	*Canmovirus*		
New taxon	Species	*Canmovirus mahaense*	Pedras lispivirus	OQ779241
New taxon	Genus	*Coroavirus*		
New taxon	Species	*Coroavirus germense*	blattodean arli-related virus OKIAV101	MT153397
New taxon	Genus	*Robevirus*		
New taxon	Species	*Robevirus hanzense*	Hángzhōu lispivirus 1	MZ209712
New taxon	Genus	*Weflthvirus*		
New taxon	Species	*Weflthvirus itaense*	Frankliniella occidentalis associated mononegavirales virus 1	MN714688

*Source/full text: https://ictv.global/ictv/proposals/2024.008M.Lispiviridae_5ngen_11nsp.zip.

## 2024.009M.Mammarenavirus_1nsp

**Title:** Create one new species in the genus *Mammarenavirus* (*Hareavirales: Arenaviridae*)

**Authors:** Shedroff ES, Martin ML, Whitmer SLM (Evk3@cdc.gov), Brignone J, Garcia JB, Sen C, Nazar Y, Fabbri C, Morales-Betoulle M, Mendez JA, Montgomery JM, Morales MA, Klena JD

**Summary**

**Taxonomic rank(s) affected**
*Hareavirales*: *Arenaviridae*: *Mammarenavirus*
**Description of current taxonomy**
Eleven genomes representing four species within American mammarenavirus clade C were present in public records. An additional 13 clade C mammarenavirus genomes were added to public records following the sequencing of mammarenavirus-positive rodent samples collected in Argentina from 1990 to 2020.
**Proposed taxonomic change(s)**
Establishment of one new species in genus *Mammarenavirus* for a virus named vello virus, identified following the sequencing of mammarenavirus-positive rodent samples collected in Argentina from 1990 to 2020.
**Justification**
Two of the L segment sequences of 13 clade C mammarenavirus genomes identified following the sequencing of mammarenavirus-positive rodent samples collected in Argentina from 1990 to 2020, meet current demarcation species criteria for the genus *Mammarenavirus*. We propose the two isolates described by Shedroff *et al*. [9] to represent a virus named ‘vello virus’ and to assign this virus to a new species, *Mammarenavirus vello*.
**Submitted:** 24/05/2024

**Table 10.** *Mammarenavirus*, one new taxon*

**Table:** 

Operation	Rank	New taxon name	Exemplar	Accession
New taxon	Species	*Mammarenavirus vello*	vello virus	L: OR844405; S: OR844394

*Source/full text: https://ictv.global/ictv/proposals/2024.009M.Mammarenavirus_1nsp.zip.

## 2024.010M.Orthobunyavirus_4nsp

**Title:** Create four new species in the genus *Orthobunyavirus*, family *Peribunyaviridae*

**Authors:** de Souza WM (wmdesouza@uky.edu), Calisher C, Carrera JP, Hughes HR, Nunes MRT, Russell B, Tilston-Lunel NL, Venter M, Xia H

**Summary**

**Taxonomic rank(s) affected**
Species
**Description of current taxonomy**
The *Peribunyaviridae* family includes 148 viral species, classified into 8 genera: *Gryffinivirus* (2 species), *Herbevirus* (3 species), *Khurdivirus* (1 species), *Lakivirus* (1 species), *Lambavirus* (1 species), *Orthobunyavirus* (134 species), *Pacuvirus* (5 species) and *Shangavirus* (1 species).
**Proposed taxonomic change(s)**
We propose the demarcation of four new species in the genus *Orthobunyavirus*.
**Justification**
Based on the current demarcation criteria of <96 % identity of L protein amino acid sequence marking a new species, we propose the demarcation of two new species in the genus *Orthobunyavirus* (*Peribunyaviridae*).
**Submitted:** 11/06/24; **Revised:** 12/08/24

**Table 11.** *Orthobunyavirus*, 4 new taxa*

**Table:** 

Operation	Rank	New taxon name	Exemplar	Accession
New taxon	Species	*Orthobunyavirus taniyamense*	Taniyama virus	S: LC698002; M: LC698003; L: LC698004
New taxon	Species	*Orthobunyavirus lichuanense*	Lichuan virus	S: MT198371; M: MT198372; L: MT198373
New taxon	Species	*Orthobunyavirus indianense*	I612045 virus	S: HM627180; M: HM627181; L: HM627182
New taxon	Species	*Orthobunyavirus taiense*	Tai orthobunyavirus	S: OQ031275; M: OQ031274; L: OQ031273

*Source/full text: https://ictv.global/ictv/proposals/2024.010M.Orthobunyavirus_4nsp.zip.

## 2024.011M.Orthohantavirus_1nsp

**Title:** Create one new species in the genus *Orthohantavirus* (*Elliovirales*: *Hantaviridae*): *Orthohantavirus ozarkense*

**Authors:** Mull N (nmull@shawnee.edu), Erdin M, Smura T, Sironen T, Forbes KM

**Summary**

**Taxonomic rank(s) affected**
*Hantaviridae*: *Orthohantavirus*
**Description of current taxonomy**
35 established species
**Proposed taxonomic change(s)**
Addition of one new species
**Justification**
Using a coding-complete genome sequence comprising all three genomic segments, we demonstrate that a virus (Ozark virus, OZAV) discovered in hispid cotton rats (*Sigmodon hispidus* Say and Ord, 1825) sampled in the Ozark Plateau, Arkansas, USA, is a genetically distinct orthohantavirus. We propose a novel species, *Orthohantavirus ozarkense*, to include OZAV.
**Submitted:** 12/05/24

**Table 12.** *Orthohantavirus*, one new taxon*

**Table:** 

Operation	Rank	New taxon name	Exemplar	Accession
New taxon	Species	*Orthohantavirus ozarkense*	Ozark virus	S: PP434897; M: PP434896; L: PP4348921

*Source/full text: https://ictv.global/ictv/proposals/2024.011M.Orthohantavirus_1nsp.zip.

## 2024.012M.Orthohantavirus_1nsp

**Title:** Create one new species in the genus *Orthohantavirus* (*Elliovirales*: *Hantaviridae*): *Orthohantavirus sagercreekense*

**Authors:** Mull N (nmull@shawnee.edu), Erdin M, Letko M, Seifert S, Sironen T, Smura T, Forbes KM

**Summary**

**Taxonomic rank(s) affected**
*Hantaviridae*: *Orthohantavirus*
**Description of current taxonomy**
35 established species
**Proposed taxonomic change(s)**
Addition of one new species
**Justification**
Using a coding-complete genome sequence comprising all three genomic segments, we demonstrate that a virus (Sager Creek virus, SACRV) discovered in prairie voles (*Microtus* (*Pedomys*) *ochrogaster* (Wagner, 1842)) sampled in the Ozark Plateau, Arkansas, USA, is a genetically unique orthohantavirus. We propose a novel species, *Orthohantavirus sagercreekense*, to include SACRV.
**Submitted:** 12/05/24

**Table 13.** *Orthohantavirus*, one new taxon*

**Table:** 

Operation	Rank	New taxon name	Exemplar	Accession
New taxon	Species	*Orthohantavirus sagercreekense*	Sager Creek virus	S: PP905729; M: PP905731; L: PP905726

*Source/full text: https://ictv.global/ictv/proposals/2024.012M.Orthohantavirus_1nsp.zip.

## 2024.013M.Phasmaviridae_4nsp_3ab_2rn

**Title:** Create four new species, abolish three species, and rename two species in the family *Phasmaviridae*

**Authors:** Ballinger MJ (ballinger@biology.msstate.edu), Junglen S, De Coninck L

**Summary**

**Taxonomic rank(s) affected**
Species
**Description of current taxonomy**
The family *Phasmaviridae* includes 29 species organized across 7 genera.
**Proposed taxonomic change(s)**
Create four new species, abolish three species established previously, and rename two species established previously.
**Justification**
Coding-complete virus genome sequences are available to justify the creation of four new species. Each exhibit 1% L protein amino acid sequence identity to other exemplar viruses in the family *Phasmaviridae*. Three species were previously established in error due to an oversight; the available genomes are not coding-complete. Two previously established species epithets erroneously referred to places and are renamed here using appropriate suffixes.
**Submitted:** 06/06/24

**Table 14.** *Phasmaviridae*, four new taxa*

**Table:** 

Operation	Rank	New taxon name	Exemplar	Accession
New taxon	Species	*Jonvirus spilikinsis*	Spilikins virus	L: MZ202269; M: MZ202270; S: MZ202271
New taxon	Species	*Jonvirus mikadosis*	Mikado virus	L: MZ202272; M: MZ202273; S: MZ202274
New taxon	Species	*Orthophasmavirus obscurae*	Drosophila North Esk phasmavirus	L: OR605709; M: OR605710; S: OR605711
New taxon	Species	*Orthophasmavirus stecellulae*	Anopheles stephensi orthophasmavirus	L: LC775043; M: LC775044; S: LC775045

**Table 15.** *Phasmaviridae*, three abolish taxa*

**Table:** 

Operation	Rank	Abolished taxon name
Abolish taxon	Species	*Feravirus hemipterus*
Abolish taxon	Species	*Orthophasmavirus flenense*
Abolish taxon	Species	*Orthophasmavirus coleopterus*

**Table 16.** *Phasmaviridae*, two rename taxa*

**Table:** 

Operation	Rank	New taxon name	Previous taxon name
Rename taxon	Species	*Orthophasmavirus miglotalis*	*Orthophasmavirus miglotasense*
Rename taxon	Species	*Orthophasmavirus barstukorius*	*Orthophasmavirus barstukasense*

*Source/full text: https://ictv.global/ictv/proposals/2024.013M.Phasmaviridae_4nsp_3ab_2rn.zip.

## 2024.014M.Platrhavirus_2ng_30nsp

**Title:** Rename the existing genus *Platrhavirus* (as *Alphaplatrhavirus*) and create 12 new species in the renamed genus, create the new genus *Betaplatrhavirus* including 12 new species and create the new genus *Gammaplatrhavirus* including 6 new species (*Mononegavirales: Rhabdoviridae*)

**Authors:** Walker PJ (peter.walker@uq.edu.au), Bejerman N, Blasdell KR, Debat H, Dietzgen RG, Fooks AR, Freitas-Astúa J, Ramos-González PL, Kondo H, Kurath G, Shi M, Tesh RB, Tordo N, Vasilakis N, Whitfield AE

**Summary**

**Taxonomic rank(s) affected**
Genus and species (*Mononegavirales*: *Rhabdoviridae*)
**Description of current taxonomy**
The family *Rhabdoviridae* currently comprises four subfamilies and one genus (*Platrhavirus*) unassigned to a subfamily that includes six species.
**Proposed taxonomic change(s)**
Rename the existing genus *Platrhavirus* (as *Alphaplatrhavirus*) and create 12 new species in the renamed genus, and create 2 new genera (*Betaplatrhavirus* and *Gammaplatrhavirus*) including 18 new species for viruses detected by metagenomic sequencing in cestode or trematode worms (Platyhelminthes) or in the faeces or visceral organs of animals (mammals, fish or crustaceans) that appear to have been infested with worms.
**Justification**
The viruses cluster phylogenetically with others in the existing or proposed genera in ML trees inferred using L protein sequences. All new species in existing genera meet established demarcation criteria. Members of the proposed renamed and new genera for viruses are well-separated phylogenetically from each other and other rhabdoviruses.
**Submitted:** 09/06/24; **Revised:** 24/08/24

**Table 17.** *Platrhavirus*, 32 new taxa*

**Table:** 

Operation	Rank	New taxon name	Exemplar	Accession
New taxon	Species	*Alphaplatrhavirus dendriticum*	Dicrocoilium rhabdo-like virus 2	OP627658
New taxon	Species	*Alphaplatrhavirus solidus*	Schistocephalus solidus rhabdovirus	MN803433
New taxon	Species	*Alphaplatrhavirus wufeng*	Wufeng shrew rhabdovirus 5	OQ715673
New taxon	Species	*Alphaplatrhavirus smithii*	Wufeng shrew rhabdovirus 7	OQ715674
New taxon	Species	*Alphaplatrhavirus chodsigoa*	Wufeng shrew rhabdovirus 8	OQ715683
New taxon	Species	*Alphaplatrhavirus hubei*	Wufeng shrew rhabdovirus 9	OQ715680
New taxon	Species	*Alphaplatrhavirus jingmen*	Jingmen bat rhabdovirus 1	OQ715681
New taxon	Species	*Alphaplatrhavirus ricketti*	Jingmen bat rhabdovirus 2	OQ715691
New taxon	Species	*Alphaplatrhavirus wenzhou*	Wenzhou bat rhabdovirus 1	OQ715676
New taxon	Species	*Alphaplatrhavirus langier*	Wenzhou bat rhabdovirus 3	OQ715675
New taxon	Species	*Alphaplatrhavirus larvatus*	rhabdovirus sp. Hlgxc14/3	OR868933
New taxon	Species	*Alphaplatrhavirus acutispina*	Wenling dimarhabdovirus 8	MG600017
New taxon	Genus	*Betaplatrhavirus*		
New taxon	Species	*Betaplatrhavirus nodulosus*	triaenorhabdovirus 2	BK059680
New taxon	Species	*Betaplatrhavirus psilotrema*	psilorhabdovirus 1	BK059745
New taxon	Species	*Betaplatrhavirus simillimum*	psilorhabdovirus 2	BK059746
New taxon	Species	*Betaplatrhavirus sphaeroidotrema*	sphaeridiorhabdovirus 2	BK059663
New taxon	Species	*Betaplatrhavirus pseudoglobulus*	sphaeridiorhabdovirus 3	BK059664
New taxon	Species	*Betaplatrhavirus himastelon*	Himastelon rhabdovirus	OR553881
New taxon	Species	*Betaplatrhavirus beihai*	Beihai dimarhabdovirus 1	MG600012
New taxon	Species	*Betaplatrhavirus wenling*	Wenling dimarhabdovirus 1	MG600014
New taxon	Species	*Betaplatrhavirus fujian*	Fujian dimarhabdovirus	MG600015
New taxon	Species	*Betaplatrhavirus fuscus*	Eptesicus fuscus rhabdovirus	MT732687
New taxon	Species	*Betaplatrhavirus abramus*	bat-associated rhabdovirus 2	OR951388
New taxon	Species	*Betaplatrhavirus armiger*	rhabdovirus sp. Hagxc131516/2	OR869044
New taxon	Genus	*Gammaplatrhavirus*		
New taxon	Species	*Gammaplatrhavirus dendriticum*	Dicrocoilium rhabdo-like virus 1	OP548620
New taxon	Species	*Gammaplatrhavirus orientalis*	metorhabdovirus 1	BK059675
New taxon	Species	*Gammaplatrhavirus sinensis*	clonorhabdovirus 1	BK059698
New taxon	Species	*Gammaplatrhavirus beihai*	Beihai barnacle virus 7	KX884411
New taxon	Species	*Gammaplatrhavirus jilin*	barnaclevirus sp.	OR871063
New taxon	Species	*Gammaplatrhavirus wenzhou*	Wenzhou bat rhabdovirus 2	OQ715697

**Table 18.** *Platrhavirus*, seven rename taxa*

**Table:** 

Operation	Rank	New taxon name	Previous taxon name
Rename taxon	Genus	*Alphaplatrhavirus*	*Platrhavirus*
Rename taxon	Species	*Alphaplatrhavirus microphallus*	*Platrhavirus microphallus*
Rename taxon	Species	*Alphaplatrhavirus nodulosis*	*Platrhavirus nodulosis*
Rename taxon	Species	*Alphaplatrhavirus orientalis*	*Platrhavirus orientalis*
Rename taxon	Species	*Alphaplatrhavirus pseudoglobulus*	*Platrhavirus pseudoglobulus*
Rename taxon	Species	*Alphaplatrhavirus turkestanicum*	*Platrhavirus turkestanicum*
Rename taxon	Species	*Alphaplatrhavirus vulpes*	*Platrhavirus vulpes*

*Source/full text: https://ictv.global/ictv/proposals/2024.014M.Platrhavirus_2ng_30nsp.zip.

## 2024.015M.Rotavirus_2nsp

**Title:** Create two new species (*Rotavirus kappagastroenteritidis, Rotavirus lambdagastroenteritidis*) in the genus *Rotavirus* (family *Sedoreoviridae*)

**Author:** Johne R (Reimar.Johne@bfr.bund.de)

**Summary**

**Taxonomic rank(s) affected**
The genus *Rotavirus*
**Description of current taxonomy**
Currently, the genus *Rotavirus* includes nine different rotavirus species.
**Proposed taxonomic change(s)**
Two new rotavirus species (*Rotavirus kappagastroenteritidis* and *Rotavirus lambdagastroenteritidis*) should be created.
**Justification**
Evolutionary analysis of complete coding regions of the novel rotavirus genomes (rotavirus K for *Rotavirus kappagastroenteritidis* and rotavirus L for *Rotavirus lambdagastroenteritidis*) indicates a separate branching on phylogenetic trees of all genome segments from those of established rotavirus species. In addition, the maximum identities of their deduced VP6 amino acid sequences with those of established rotavirus species reference strains are 51% for rotavirus K and 47% for rotavirus L, which are lower than the cut-off value (53%) for the definition of new *Rotavirus* species.
**Submitted:** 14/06/2024

**Table 19.** *Rotavirus*, two new taxa*

**Table:** 

Operation	Rank	New taxon name	Exemplar	Accession
New taxon	Species	*Rotavirus kappagastroenteritidis*	rotavirus K	OQ934016; OQ934017; OQ934018; OQ934019; OQ934020; OQ934021; OQ934022; OQ934023; OQ934024; OQ934025; OQ934026
New taxon	Species	*Rotavirus lambdagastroenteritidis*	rotavirus L	OM101015; OM101016; OM101017; OM101018; OM101019; OM101020; OM101021; OM101022; OM101023; OM101024; OM101025

*Source/full text: https://ictv.global/ictv/proposals/2024.015M.Rotavirus_2nsp.zip.

## 2024.016M.Sedoreoviridae_1ng_3nsp

**Title:** Create one new genus (*Crabreovirus*) with three new species

**Authors:** Zhao M (mzhao@rvc.ac.uk), Schott E (schott@umces.edu)

**Summary**

**Taxonomic rank(s) affected**
*Sedoreoviridae* family
**Description of current taxonomy**
*Sedoreoviridae* currently has six genera, namely *Cardoreovirus*, *Mimoreovirus*, *Orbivirus*, *Phytoreovirus*, *Rotavirus* and *Seadornavirus*.
**Proposed taxonomic change(s)**
A new genus, named *Crabreovirus*, should be established in the *Sedoreoviridae* family. This new genus should include three new species, including *Crabreovirus callinectes*, *Crabreovirus scylla* and *Crabreovirus eriocheiris*.
**Justification**
Three representative viruses of the proposed new *Crabreovirus* genus exhibit less than 20% amino acid sequence identity in VP1 when compared to virus members of other established genera within the *Sedoreoviridae* family. A maximum likelihood phylogenetic tree shows that viruses in the three proposed new species form a distinct clade from members of other *Sedoreoviridae* genera, yet remain within the same clade as each other. The phylogenetic analysis supports the classification of these three species into a new genus, *Crabreovirus*.
**Submitted:** 14/06/24

**Table 20.** *Sedoreoviridae*, four new taxa*

**Table:** 

Operation	Rank	New taxon name	Exemplar	Accession
New taxon	Genus	*Crabreovirus*		
New taxon	Species	*Crabreovirus callinectes*	Callinectes sapidus reovirus 1	KU311708; KU311709; KU311710; KU311711; KU311712; KU311713; KU311714; KU311715; KU311716; KU311717; KU311718: KU311719
New taxon	Species	*Crabreovirus scylla*	Scylla serrata reovirus SZ-2007	HQ414127; HQ414128; HQ414129; HQ414130; HQ414131; HQ414132; HQ414133; HQ414134; HQ414135; HQ414136; HQ414137; HQ414138
New taxon	Species	*Crabreovirus eriocheiris*	Eriocheir sinensis reovirus WX-2012	KP638402; KP638403; KP638404; KP638405; KP638406; KP638407; KP638408; KP638409; KP638410; KP638411; KP638412; KP638413

*Source/full text: https://ictv.global/ictv/proposals/2024.016M.Sedoreoviridae_1ng_3nsp.zip.

## 2024.017M.Shilevirus_10nsp

**Title:** Create ten new species in genus *Shilevirus* (*Bunyaviricetes*: *Hareavirales*: *Leishbuviridae*)

**Authors:** Yurchenko, V (Vyacheslav.Yurchenko@osu.cz), Kuhn, JH, Kostygov, AY

**Summary**

**Taxonomic rank(s) affected**
Leishbuvirid genus *Shilevirus*
**Description of current taxonomy**
One species (*Shilevirus leptomonadis*)
**Proposed taxonomic change(s)**
Add 10 new species
**Justification**
Discovery of novel shileviruses in various hosts from various habitats with sufficient genetic divergence.
**Submitted:** 21/06/24

**Table 21.** *Shilevirus*, 10 new taxa*

**Table:** 

Operation	Rank	New taxon name	Exemplar	Accession
New taxon	Species	*Shilevirus alphablechomonadis*	Blechmonas luni leishbunyavirus 1	S: MG967336; M: MG967335; L: MG967334
New taxon	Species	*Shilevirus betablechomonadis*	Blechomonas ayalai leishbunyavirus 1	S: MG967340; M: MG967339; L: MG967338
New taxon	Species	*Shilevirus puertonapoense*	Crithidia abscondita leishbunyavirus	S: KX507299; M: KX507300; L: KX507301
New taxon	Species	*Shilevirus crithidiaebombi*	Crithidia bombi leishbuvirus 1	S: OR146998; M: OR146997; L: OR146996
New taxon	Species	*Shilevirus otongatchiense*	Crithidia otongatchiensis leishbunyavirus	S: KX451144; M: KX683300; L: KX451145
New taxon	Species	*Shilevirus alphamoraviense*	Leptomonas pyrrhocoris leishbunyavirus 3	S: OP722879; M: OP722878; L: OP722877
New taxon	Species	*Shilevirus betamoraviense*	Leptomonas pyrrhocoris leishbunyavirus 4	S: OP722876; M: OP722875; L: OP722874
New taxon	Species	*Shilevirus martiniquense*	Leishmania martiniquensis leishbunyavirus 1	S: MK356556; M: MK356555; L: MK356554
New taxon	Species	*Shilevirus moramangoense*	Leptomonas moramango leishbunyavirus isolate LepmorLBV1b	S: KX280017; M: KX280016; L: KX280015
New taxon	Species	*Shilevirus gammablechomonadis*	Blechomonas maslovi leishbunyavirus 1	S: MG967344; M: MG967343; L: MG967342

*Source/full text: https://ictv.global/ictv/proposals/2024.017M.Shilevirus_10nsp.zip.

## 2024.018M.Tosoviridae_move

**Title:** Move free-floating negarnaviricot family *Tosoviridae* into the bunyaviricete order *Hareavirales*

**Authors:** Kuhn JH, Koonin EV, Krupovic M, Wolf Y (wolf@ncbi.nlm.nih.gov)

**Summary**

**Taxonomic rank(s) affected**
Family (*Tosoviridae*)
**Description of current taxonomy**
*Riboviria*: *Orthornavirae*: *Negarnaviricota*: *Tosoviridae*
**Proposed taxonomic change(s)**
Move family *Tosoviridae* to order *Hareavirales* (*Polyploviricotina*: *Bunyaviricetes*)
**Justification**
Updated RdRP phylogeny unambiguously groups tosovirids with hareavirals (sister to hareaviral families *Nairoviridae* and *Wupedeviridae*)
**Submitted:** 21/06/24; **Revised:** 16/08/24

**Table 22.** *Tosoviridae*, one move taxon*

**Table:** 

Operation	Rank	Taxon name	Old parent taxon	New parent taxon
Move taxon	Family	*Tosoviridae*	*Negarnaviricota*	*Hareavirales*

*Source/full text: https://ictv.global/ictv/proposals/2024.018M.Tosoviridae_move.zip.

## Supplementary material

10.1099/jgv.0.002112 Supplementary Material 1.

## Abbreviations

ICTV: International Committee on Taxonomy of Viruses
RdRP: RNA-directed RNA polymerase
SC: Subcommittee
